# Tracking maize pollen development by the *Leaf Collar Method*

**DOI:** 10.1007/s00497-017-0311-4

**Published:** 2017-11-04

**Authors:** Kevin Begcy, Thomas Dresselhaus

**Affiliations:** Cell Biology and Plant Biochemistry, Biochemie-Zentrum Regensburg, University of Regensburg, 93053 Regensburg, Germany

**Keywords:** Maize, Pollen development, Mitosis, Meiosis, Sperm cell, Nondestructive method

## Abstract

*****Key message***:**

**An easy and highly reproducible nondestructive method named the Leaf Collar Method is described to identify and characterize the different stages of pollen development in maize.**

**Abstract:**

In plants, many cellular events such as meiosis, asymmetric cell division, cell cycle regulation, cell fate determination, nucleus movement, vacuole formation, chromatin condensation and epigenetic modifications take place during pollen development. In maize, pollen development occurs in tassels that are confined within the internal stalk of the plant. Hence, identification of the different pollen developmental stages as a tool to investigate above biological processes is impossible without dissecting the entire plant. Therefore, an efficient and reproducible method is necessary to isolate homogeneous cell populations at individual stages throughout pollen development without destroying the plant. Here, we describe a method to identify the various stages of pollen development in maize. Using the *Leaf Collar Method* in the maize inbreed line B73, we have determined the duration of each stage from pollen mother cells before meiosis to mature tricellular pollen. Anther and tassel size as well as percentage of pollen stages were correlated with vegetative stages, which are easily recognized. The identification of stage-specific genes indicates the reproducibility of the method. In summary, we present an easy and highly reproducible nondestructive method to identify and characterize the different stages of pollen development in maize. This method now opens the way for many subsequent physiological, morphological and molecular analyses to study, for instance, transcriptomics, metabolomics, DNA methylation and chromatin patterns during normal and stressful conditions throughout pollen development in one of the economically most important grass species.

**Electronic supplementary material:**

The online version of this article (10.1007/s00497-017-0311-4) contains supplementary material, which is available to authorized users.

## Introduction

The dissection of male gametophyte (pollen) development provides a unique tool to study many fundamental questions in plant cell biology and reproduction. Moreover, microspores as one stage during pollen development represent the major source to generate di-haploid plants and thus are key to modern agriculture. The events that culminate in the formation and release of mature pollen grains from anthers initiate with the formation of diploid pollen mother cells followed by meiosis generating four microspores, which further develop into pollen grains harboring one and two haploid sperm, respectively, enclosed by the vegetative tube cell (Figure S1). Pollen development in anthers involves intricate and tightly controlled sets of structural and molecular changes and is characterized by a relatively synchronized gene expression pattern (Rutley and Twell 2015). Although the importance of knowing the precise developmental stages of crop plants has been recognized for many years (Leather 2010), stages of pollen development have been correlated with plant age rather than growth stages. Previous reports have also shown a correlation between anther length and pollen development (Chang and Neuffer 1989), but plants had to be dissected to isolate anthers. Thus, implementing an effective method of correlating vegetative plant development with pollen stages is fundamental for an accurate selection of material for gene expression analyses, comparative genomics and physiology, as well as investigations of pollen development under stress.

Maize (*Zea mays*) has been proposed as a model to study reproductive development in grasses and monocots in general (Dresselhaus et al. 2011). Several cellular and genetic tools, including the availability of transposon insertion lines and CRISPR/Cas9 gene editing, are available making maize plants an attractive organism to be studied. Within the extensive maize populations generated over the past centuries, B73 maize inbred line is one of the most widely used varieties and represents the line whose genome has been sequenced (Schnable et al. 2009) and which now serves as a maize reference genome. In conclusion, B73 provides an excellent tool for further genomic studies.

The most accessible developmental stages of pollen are mature pollen grains and pollen tubes, and these are consequently the stages most extensively studied in various plant species (Rutley and Twell 2015). In contrast, only few studies have focused on the synchronized pollen developmental process (Bedinger and Edgerton 1990; Dukowic-Schulze et al. 2014; Ma et al. 2008), mainly due to difficulties of accurately determining pollen stages and sample collection. Maize pollen development occurs within the florets of the tassel, which is confined in the stalk. Therefore, studies involving developmental progression of reproductive structures become a difficult task due to sampling inaccessibility without destroying the plant. The emerging tassel is formed within the plant stem, which is surrounded by newly folded blades and sheaths that protect it (Bonnett 1954). This important peculiarity makes the precise monitoring of pollen development and access to the tassel particularly difficult without dissecting the entire plant. Thus, it is essential to identify an external vegetative trait that is accurate, reproducible and easily recognizable to monitor and isolate different stages of maize pollen development.

**Fig. 1:**
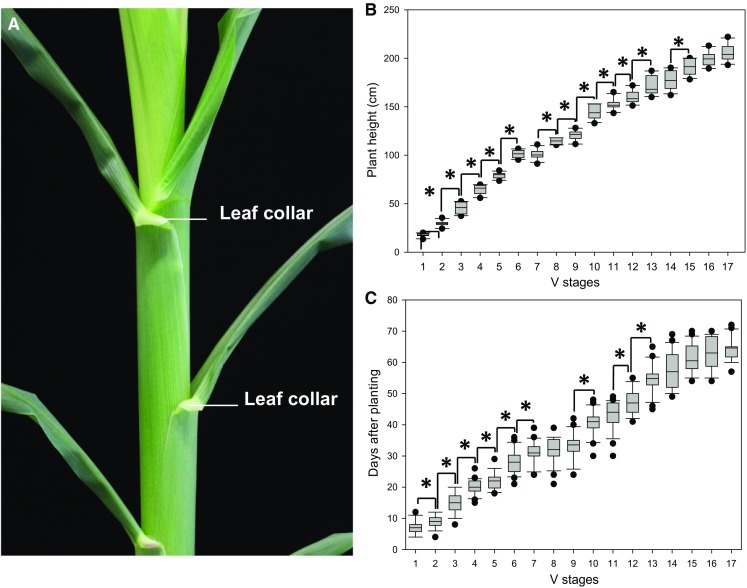
Maize development determined by the *Leaf Collar Method*. **a** Leaf collar structures on the base of maize leaves. **b** Developmental stages and their respective plant height of maize inbred line B73. **c** Vegetative developmental stages (V-stages) according to Abendroth et al. (2011) after days of planting. (*) Indicates significant differences relative to the previous developmental stage at *P* < 0.001. Dots plotted represent outliers of the interquartile range of the individual measurements. Error bars represent SD (*n* = 5 plants for each V-stage) in at least five independent experiments

Zadoks decimal code is the most frequently used external system to determine cereals development (Zadoks et al. 1974). In wheat, rice and barley, adaptations to the original scale have been carried out (Chin et al. 1991; Landes and Porter 1989; Tottman 1987; Zadoks et al. 1974). However, this popular staging method does not allow to accurately determine pollen development. In maize, the most widely used method to determine vegetative stages is the *Leaf Collar Method*. This method determines the leaf stage in maize by counting the number of leaves on a plant with visible leaf collars, beginning with the lowermost, short, rounded-tip true leaf and ending with the uppermost leaf with a visible leaf collar (Abendroth et al. 2011). The leaf collar is the light-colored collar-like band located at the base of an exposed leaf blade, near the spot where the leaf blade comes into contact with the stem of the plant and from where it extends away from the stalk (Fig. 1a).

We report here a nondestructive method to determine pollen development stages in maize. The method allows to precisely isolate large quantities of pollen for further studies. Moreover, since this pollen development prediction analysis is based upon easily defined growth characteristics or vegetative (V) stages, this method can also be easily adapted to other maize varieties and other growth conditions.

## Materials and methods

### Plant material and growth conditions

Seeds of the maize inbred line B73 were germinated in an incubator and then transferred to pots (10 cm diameter, 10 seedlings per pot) containing a substrate and soil mixture (1:1, v/v). Maize B73 seedlings were then transferred to 10-L pots to the greenhouse under controlled conditions of 14 h of light at 25 ± 2 °C and 10 h of darkness at 21 ± 2 °C and a constant air humidity of 60–65%. Supplemented light of 16,000 LUX was provided to adjust day length duration. An automated temperature-water-based irrigation system was used to supply water according to plant consumption in a time-based pre-programmed schedule. Plants were fertilized twice a week with 2% Hakaphos (Compo Expert) and monitored throughout their entire vegetative and reproductive development.

### Leaf Collar Method

Vegetative (V) stages were determined by the total number of leaves with visible collars, for instance, plants with seven visible leaf collars are at V7. A collar structure is the off-white band at the base of the leaf blade where it extends away from the stalk. During plant development, an increase in plant size and number of leaves was observed; however, lower leaves are naturally damaged and lost. Therefore, it is important to count them since the beginning; otherwise, developmental stages will be misidentified, generating errors determining V-stages. Leaves within the whorl, not yet fully expanded and with no visible leaf collar are not included in this leaf staging method (Abendroth et al. 2011). After plants reached selected V-stages, plants were dissected and pollen development was monitored.

### Male gametophyte isolation and visualization

Pollen grains isolated at individual V-stages were used to determine their male gametophyte stages. To determine developmental stages, pollen was mounted on glass slides containing 1 mg/mL DAPI solution in 1X PBS (0.8% NaCl; 0.002% KCl; 0.014% of Na_2_HPO_4_; 0.0024% of KH_2_PO_4_), covered with a cover slip, sealed and observed after 5 min in a Zeiss microscope equipped for structured illumination microscopy (Apotome) and with a Zeiss AxioCam MRm camera. DAPI was excited with a filter set of 359 nm. Images were recorded using AxioCam cameras (MrC; Zeiss). Approximately, 20–25 plants were used per pollen developmental stage identification. Isolation of pollen developmental stages was followed by the capillary collection of meiocytes methods already described in maize (Dukowic-Schulze et al. 2014).

### RNA isolation and qPCR

Total pollen RNA was isolated using RNA plant mini kit (Ambion) following the manufacturer’s instructions. cDNA synthesis was performed using reverse transcription system (Invitrogen SuperScript II) and oligo(dT) primers. Real-time PCR reactions were performed using KAPA SYBR Fast qPCR Master Mix (Peqlab Biotechnologie) as described by (Begcy and Walia 2015). Ubiquitin (GRMZM2G102471) was used as the internal normalization control. qPCRs were performed on the Mastercycler Realplex^2^ (Eppendorf) in a 96-well reaction plate according to the manufacturer’s recommendations. The primers that have been used are described in Table S1. Cycling parameters consisted of 5 min at 95 °C, and 40 cycles of 95 °C for 15 s, 60 °C for 30 s and 70 °C for 30 s. qPCR reactions were performed in triplicate for each RNA sample on at least three biological replicates of every pollen developmental stage. Specificity of the amplifications was verified by a melting curve analysis to test product specificity. Results from the Mastercycler Realplex^2^ detection system were further analyzed using Microsoft Excel. Relative amounts of mRNA were calculated from threshold points (Ct values) located in the log-linear range of real-time PCR amplification plots using the 2-ΔCt method.

### Statistical analysis

Statistical analyses were performed using the R software/environment. Data from at least five independent experiments, where each experiment had at least *n* = 5 for each V-stage (resulting in *n* = 25 per V-stage), were used. Data were expressed as mean, median, minimum value (min) and maximum value (max), and *p* value of 0.05 was used as the significance level.

## Results and discussion

### Variation of plant height and vegetative (V) stage transitions of maize plants staged by the Leaf Collar Method

Maize inbred line B73 was used to establish a correlation between vegetative stages and pollen development. One of the most widely used agronomical methods to characterize vegetative stages in maize is the *Leaf Collar Method* (Abendroth et al. 2011). The total number of leaves with visible leaf collars is used in this method as an indicator of vegetative (V) stages. While the *Leaf Collar Method* is used for the identification of vegetative stages, reproductive developmental stages are characterized based on visual indicators of flower and kernel development (Abendroth et al. 2011). Thus, it was unclear whether the *Leaf Collar Method* could also be used to provide appropriate categorization of early reproductive developmental stages. Therefore, we investigated whether the method could be used to accurately and reproducibly determine individual stages for maize pollen development.

We first studied plant height, which is one of the most variable traits in maize. It is well known that maize plants cultivated at the same location with similar environmental conditions display variable plant height. The correlation between maize development and temperature is also well documented (Abendroth et al. 2011). Even though we observed statistical differences among discrete V-stages, a similar plant height can be found at different V-stages indicating an unpredictability when using plant height to determine V-stages (Fig. 1b). Additionally, although early V-stages correlated well with days after planting, stages after V6–V7, where male gametophyte development is initiated, showed a large overlap and variability (Fig. 1c). For instance, when comparing plants at the same day after planting they are encountered at different V-stages, indicating a low accuracy for pollen developmental stages prediction.

### Tracking maize pollen developmental stages by the Leaf Collar Method

**Fig. 2:**
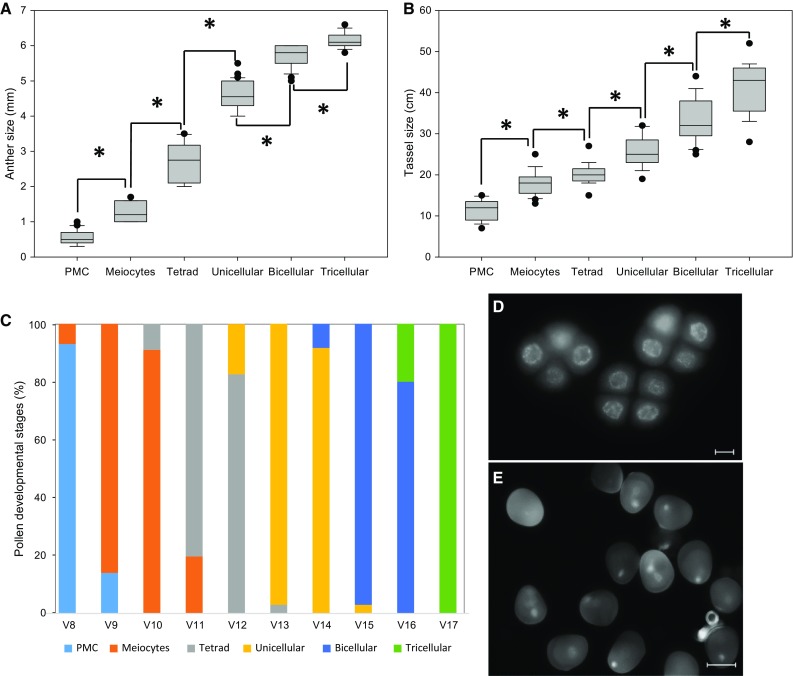
Pollen developmental stages of maize inbred line B73. **a** Stages in relation to anther, **b** tassel size and **c** vegetative development (V) stages. Average percentages of isolated/pooled cells of V-stages of maize pollen development are shown in (**c**). **d** Isolated tetrad stages at V12 stage; bar = 10 µm. **e** Pooled sample at the unicellular and bicellular stage isolated at V14 stage; bar = 50 µm. (*) Indicates significant differences relative to the previous developmental stage at *P* < 0.001. Dots plotted represent outliers of the interquartile range of the individual measurements. Error bars represent SD (*n* = 5 plants for each V-stage) in at least five independent experiments

Previous reports have shown already a correlation between anther length and pollen development (Chang and Neuffer 1989). We specifically measured anthers and tassel size in correlation with pollen developmental stages collected following the *Leaf Collar Method* (Fig. 2a). We found that our staging method allows for reliable identification of pollen development based on anther and tassel size. Even though a positive correlation among anthers and tassel size and pollen development was found (Fig. 2b), the measurements showed high variations at all stages indicating a large overlap among different developmental stages, thus inducing a low accuracy for pollen stage prediction. Furthermore, since anthers and tassels grow inside of the stalk it is particularly difficult to identify the developmental stages without dissecting maize plants. In summary, > 90% of pollen isolated at stage V8 are PMCs, about 90% at stages V9–V10 are meiocytes, tetrads occur at around 80% at stages V11–V12 and > 90% unicellular microspores at stages V13–V14. We suggest to use stage V15 to isolate > 90% bicellular pollen und V17 to isolate exclusively mature pollen (Fig. 2c). Figure 2d, e shows examples of tetrads isolated at stage V12 and a mixture of uni- and bicellular pollen at stage V14. Even though this method can predict pollen stages with high accuracy (Fig. 2c), we observed that all stages contained a small portion (5–20%) of cells containing a second pollen stage. This is due to the fact that neighboring spikelets in general exhibit highly synchronous anther and pollen development in upper florets, while development in the lower florets is about 1 day delayed (Cacharron et al. 1999; Kiesselbach 1949).

**Fig. 3:**
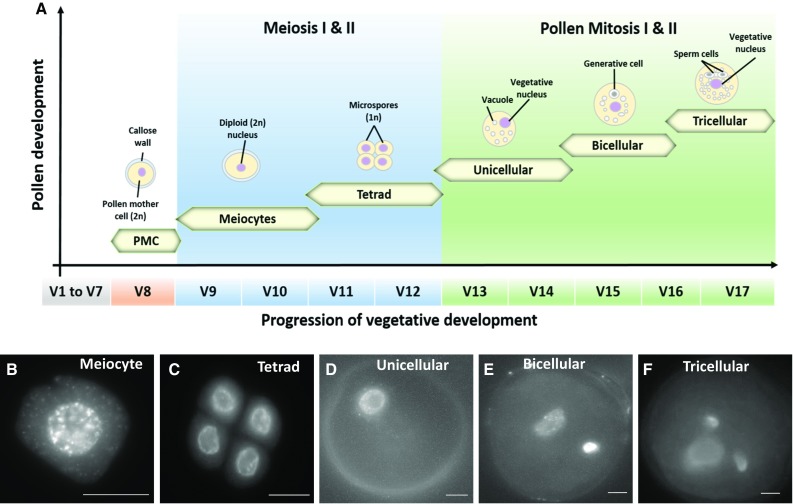
Pollen development of the maize inbred line B73 tracked by the *Leaf Collar Method*. **a** Schematic representation of the progression of vegetative stages during maize pollen development and their respective pollen developmental stages. **b–f** Pollen stages as indicated. Bars = 10 µm

Development of pollen grains (Fig. 3a) is initiated in the anthers located in the florets of a maize tassel. Four loculi in each anther contain large numbers of synchronously developing pollen. The initial step of pollen formation occurs with the development of free diploid pollen mother cells (PMCs), which predominately occurs at stage V8 (Fig. 3a and Fig. S1). Subsequent steps involve the maturation of PMCs into meiocytes (Fig. 3b), which undergo two meiotic divisions leading to the formation of tetrads (Fig. 3c) each containing four haploid microspores. This process takes place between late V8 and the beginning of V11. Next, unicellular pollen or microspores are released from tetrads and increase in size between stages V13 and V14 (Fig. 3d). Next, an asymmetric mitotic cell division (pollen mitosis I; PMI) occurs, giving rise to a small generative cell and a large vegetative cell surrounded by a thick, strongly sculptured cell wall. This stage occurring at stage V15 to early V16 is named as bicellular pollen (Fig. 3e). A subsequent second mitotic division (pollen mitosis II; PMII) of the generative cell generates two spindle-shaped sperm cells ultimately forming together with the tube cell the tricellular mature pollen grain (Fig. 3f). Mature pollen can be found after stage V17.

### Pollen stage-specific analysis of gene expression

**Fig. 4:**
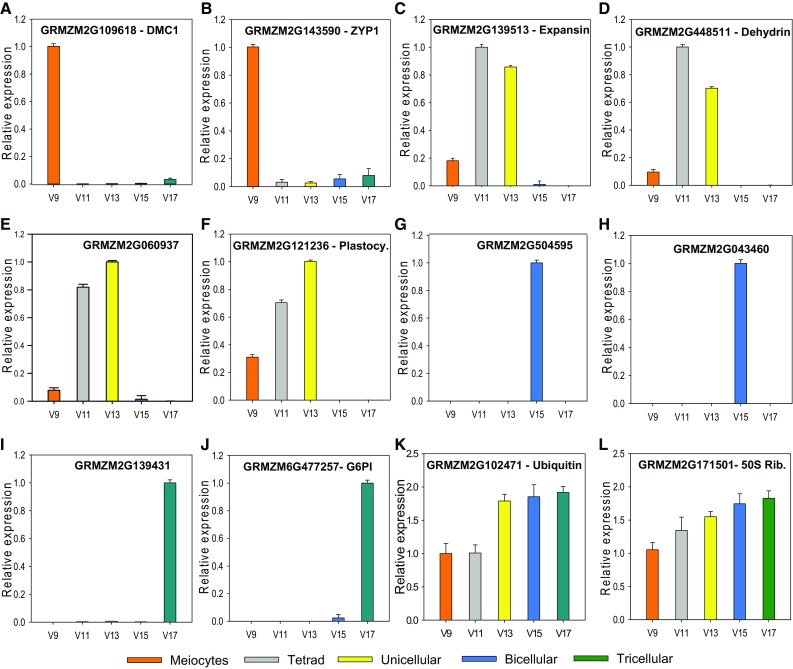
Stage-specific expression of genes during pollen development in maize. **a–b** Expression of meiocytes genes as well as **c–f** genes preferentially expressed at tetrad/unicellular (microspore) stage, **g–h** bicellular and **i–j** mature tricellular pollen stage. **k–l** Ubiquitin and 50S ribosomal protein gene L12 are shown as examples of genes expressed throughout pollen development. Gene identifiers are provided for all example genes shown

To further validate the accuracy of the method reported, we selected a list of pollen-enriched candidate genes already reported in maize and rice (Dukowic-Schulze et al. 2014; Wu et al. 2014) as well as others found in our own RNAseq data from pollen (Begcy and Dresselhaus, unpublished). A total of 12 candidate genes were used to test their expression pattern during pollen development. Two genes described as particularly expressed during meiosis in maize were selected for the confirmation of the accurate isolation of meiocytes (Dukowic-Schulze et al. 2014), the genes of meiotic recombination protein Dmc1 (GRMZM2G109618) and Zyp1 (GRMZM2G143590), a central element of the synaptonemal complex. Transcript levels of *Dmc1* and *Zyp1* were specifically found in isolated meiocytes (Fig. 4a–b). At the tetrad and unicellular pollen stage, an expansin and a dehydrin gene were found to be preferentially expressed (Fig. 4c–d). An uncharacterized gene (GRMZM2G060937) and a plastocyanin-like gene (GRMZM2G121236) were highly expressed in unicellular pollen and switched off at the bicellular stage (Fig. 4e–f). Two uncharacterized genes (GRMZM2G504595 and GRMZM2G043460) showed particularly high expression in bicellular pollen (Fig. 4g–h). Glucose-6-phosphate isomerase (GRMZM6G477257) and another uncharacterized gene (GRMZM2G139431) were almost exclusively expressed in mature pollen (Fig. 4i–j). Two constitutively expressed genes encoding ubiquitin (GRMZM2G102471) and a 50S ribosomal protein L12 (GRMZM2G171501) are shown to be present at comparable levels in all pollen samples (Fig. 4k–l).

## Conclusions

Our investigations showed that plant height and age are not accurate measures to determine pollen developmental stages. As reported earlier, anther and tassel length and pollen development (Chang and Neuffer 1989) are strongly correlated. As a vegetative noninvasive measure, we could show that the *Leaf Collar Method* can be used as a tool for identifying very pure stages of pollen development in maize with high accuracy and without damaging the plants for stage determination. This method can now be widely applied to study, for instance, transcriptome, methylome and metabolome of the various pollen stages and for functional analyses of mutants. Pollen stages such as microspores can also be easily isolated at large quantities for breeding purposes such as haploid production. Small adaptations of the method might be necessary to isolate pollen stages from other maize inbred lines.

### **Author contribution statement**

KB and TD designed the study. KB performed the experiments. KB and TD wrote and approved the final manuscript.

## Electronic supplementary material

Below is the link to the electronic supplementary material.

Supplementary material 1 (PPTX 82 kb)

## References

[CR1] Abendroth LJ, Elmore RW, Boyer MJ, Marlay SK (2011). Corn growth and development.

[CR2] Bedinger PA, Edgerton MD (1990). Developmental staging of maize microspores reveals a transition in developing microspore proteins. Plant Physiol.

[CR3] Begcy K, Walia H (2015). Drought stress delays endosperm development and misregulates genes associated with cytoskeleton organization and grain quality proteins in developing wheat seeds. Plant Sci.

[CR4] Bonnett OT (1954). The inflorescences of maize. Science.

[CR5] Cacharron J, Saedler H, Theissen G (1999). Expression of MADS box genes ZMM8 and ZMM14 during inflorescence development of Zea mays discriminates between the upper and the lower floret of each spikelet. Dev Genes Evol.

[CR6] Chang MT, Neuffer MG (1989). Maize microsporogenesis. Genome.

[CR7] Chin KM, Leohken A, Sozzi D, Williams RJ (1991). A modified Zadoks decimal code for the growth stages of rice. Trop Pest Manag.

[CR8] Dresselhaus T, Lausser A, Marton ML (2011). Using maize as a model to study pollen tube growth and guidance, cross-incompatibility and sperm delivery in grasses. Ann Bot.

[CR9] Dukowic-Schulze S, Sundararajan A, Mudge J, Ramaraj T, Farmer AD, Wang M, Sun Q, Pillardy J, Kianian S, Retzel EF, Pawlowski WP, Chen C (2014). The transcriptome landscape of early maize meiosis. BMC Plant Biol.

[CR10] Kiesselbach TA (1949). The structure and reproduction of corn.

[CR11] Landes A, Porter JR (1989). Comparison of scales used for categorizing the development of wheat, barley, rye and oats. Ann Appl Biol.

[CR12] Leather SR (2010). Precise knowledge of plant growth stages enhances applied and pure research. Ann Appl Biol.

[CR13] Ma J, Skibbe DS, Fernandes J, Walbot V (2008). Male reproductive development: gene expression profiling of maize anther and pollen ontogeny. Genome Biol.

[CR14] Rutley N, Twell D (2015). A decade of pollen transcriptomics. Plant Reprod.

[CR15] Schnable PS, Ware D, Fulton RS, Stein JC, Wei FS (2009). The B73 maize genome: complexity, diversity, and dynamics. Science.

[CR16] Tottman DR (1987). The decimal code for the growth-stages of cereals, with illustrations. Ann Appl Biol.

[CR17] Wu J, Shahid MQ, Guo H, Yin W, Chen Z, Wang L, Liu X, Lu Y (2014). Comparative cytological and transcriptomic analysis of pollen development in autotetraploid and diploid rice. Plant Reprod.

[CR18] Zadoks JC, Chang TT, Konzak CF (1974). A decimal code for the growth stages of cereals. Weed Res.

