# Supplementary material for: Tracking maize pollen development by the Leaf Collar Method
Source: Plant Reprod. 2017 Nov 4;30(4):171–8. doi: 10.1007/s00497-017-0311-4 (PMC5701949; doi:10.1007/s00497-017-0311-4)
Supplement: Supplementary file 1 — Supplementary material 1 (PPTX 82 kb) [file 497_2017_311_MOESM1_ESM.pptx]

## Slide 1
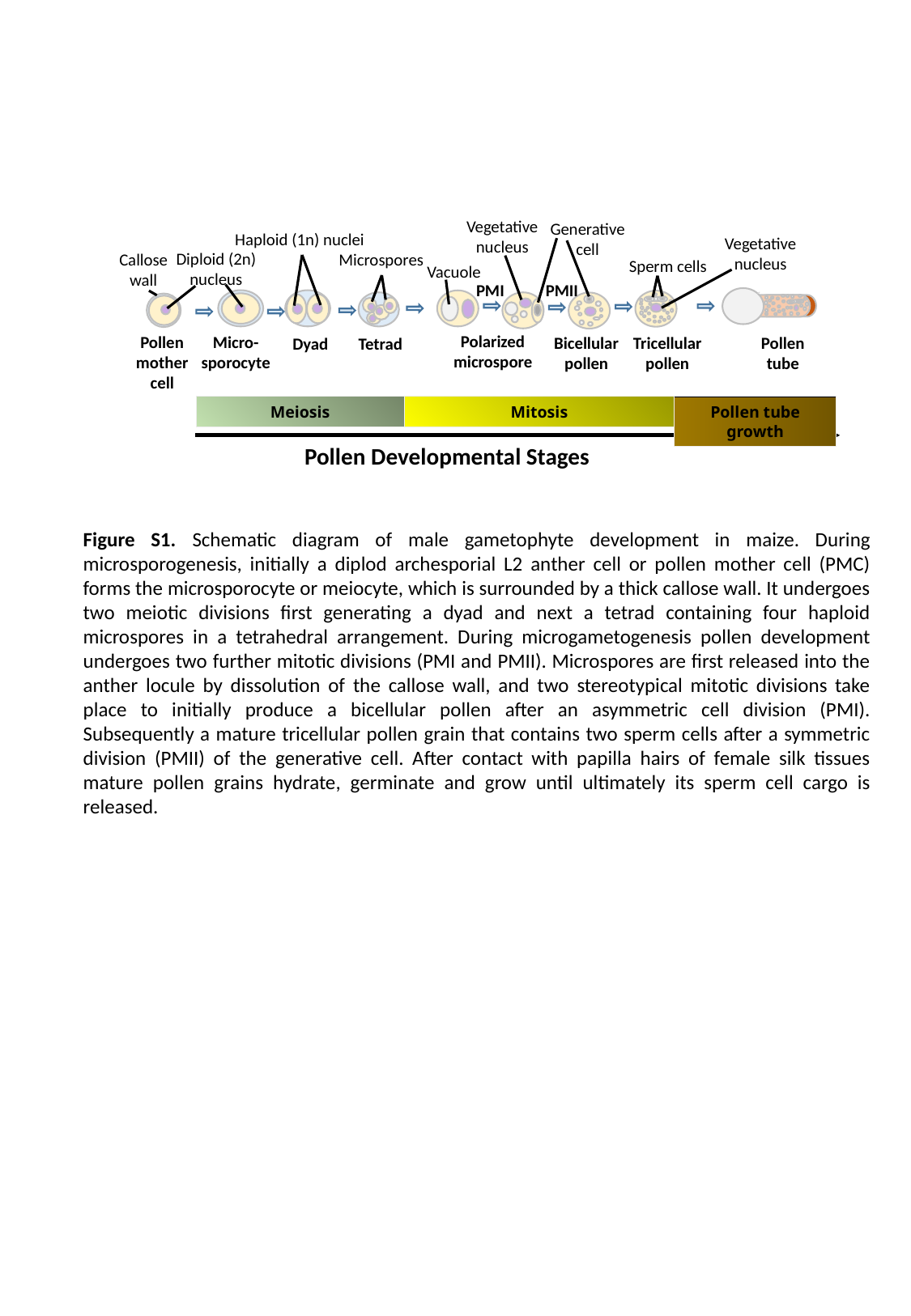

Vegetative nucleus
Generative cell
Haploid (1n) nuclei
Vegetative nucleus
Diploid (2n) nucleus
Callose wall
Microspores
Sperm cells
Vacuole
PMI
PMII
Polarized microspore
Pollen mother cell
Micro-
sporocyte
Bicellular pollen
Tricellular pollen
Pollen tube
Dyad
Tetrad
Pollen tube growth
Meiosis
Mitosis
Pollen Developmental Stages
Figure S1. Schematic diagram of male gametophyte development in maize. During microsporogenesis, initially a diplod archesporial L2 anther cell or pollen mother cell (PMC) forms the microsporocyte or meiocyte, which is surrounded by a thick callose wall. It undergoes two meiotic divisions first generating a dyad and next a tetrad containing four haploid microspores in a tetrahedral arrangement. During microgametogenesis pollen development undergoes two further mitotic divisions (PMI and PMII). Microspores are first released into the anther locule by dissolution of the callose wall, and two stereotypical mitotic divisions take place to initially produce a bicellular pollen after an asymmetric cell division (PMI). Subsequently a mature tricellular pollen grain that contains two sperm cells after a symmetric division (PMII) of the generative cell. After contact with papilla hairs of female silk tissues mature pollen grains hydrate, germinate and grow until ultimately its sperm cell cargo is released.

## Slide 2
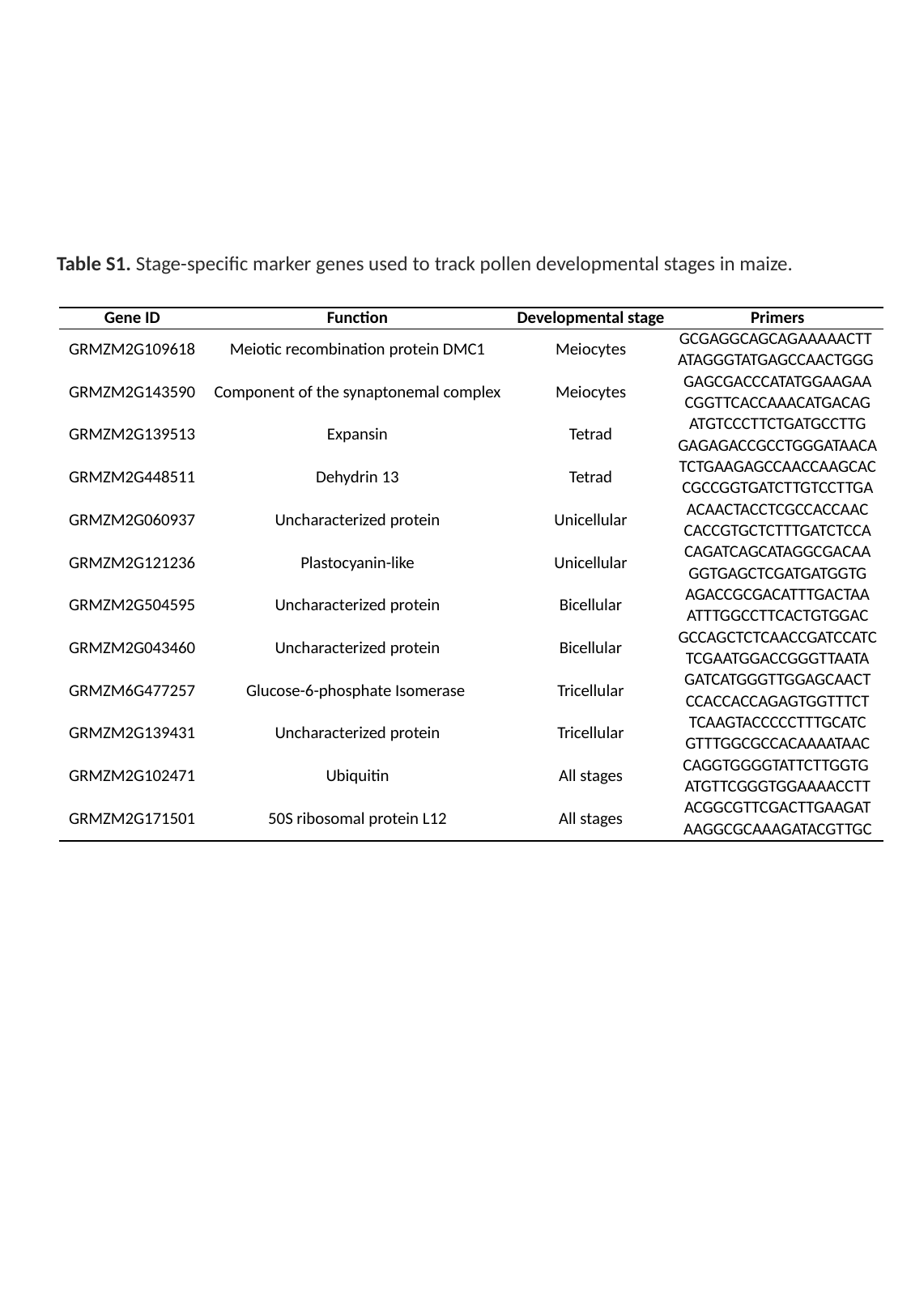

Table S1. Stage-specific marker genes used to track pollen developmental stages in maize.
| Gene ID | Function | Developmental stage | Primers |
| --- | --- | --- | --- |
| GRMZM2G109618 | Meiotic recombination protein DMC1 | Meiocytes | GCGAGGCAGCAGAAAAACTT |
| | | | ATAGGGTATGAGCCAACTGGG |
| GRMZM2G143590 | Component of the synaptonemal complex | Meiocytes | GAGCGACCCATATGGAAGAA |
| | | | CGGTTCACCAAACATGACAG |
| GRMZM2G139513 | Expansin | Tetrad | ATGTCCCTTCTGATGCCTTG |
| | | | GAGAGACCGCCTGGGATAACA |
| GRMZM2G448511 | Dehydrin 13 | Tetrad | TCTGAAGAGCCAACCAAGCAC |
| | | | CGCCGGTGATCTTGTCCTTGA |
| GRMZM2G060937 | Uncharacterized protein | Unicellular | ACAACTACCTCGCCACCAAC |
| | | | CACCGTGCTCTTTGATCTCCA |
| GRMZM2G121236 | Plastocyanin-like | Unicellular | CAGATCAGCATAGGCGACAA |
| | | | GGTGAGCTCGATGATGGTG |
| GRMZM2G504595 | Uncharacterized protein | Bicellular | AGACCGCGACATTTGACTAA |
| | | | ATTTGGCCTTCACTGTGGAC |
| GRMZM2G043460 | Uncharacterized protein | Bicellular | GCCAGCTCTCAACCGATCCATC |
| | | | TCGAATGGACCGGGTTAATA |
| GRMZM6G477257 | Glucose-6-phosphate Isomerase | Tricellular | GATCATGGGTTGGAGCAACT |
| | | | CCACCACCAGAGTGGTTTCT |
| GRMZM2G139431 | Uncharacterized protein | Tricellular | TCAAGTACCCCCTTTGCATC |
| | | | GTTTGGCGCCACAAAATAAC |
| GRMZM2G102471 | Ubiquitin | All stages | CAGGTGGGGTATTCTTGGTG |
| | | | ATGTTCGGGTGGAAAACCTT |
| GRMZM2G171501 | 50S ribosomal protein L12 | All stages | ACGGCGTTCGACTTGAAGAT |
| | | | AAGGCGCAAAGATACGTTGC |
